# O01 Adult onset PIMS-TS with secondary haemophagocytic lymphistiocytosis: into the eye of the cytokine storm

**DOI:** 10.1093/rap/rkaa053

**Published:** 2020-11-03

**Authors:** Luke Flower, Aislinn Gale, Eman Elfar, Jessica Manson, Rachel Tattersal, Vanessa Quick

**Affiliations:** r1 University College London Hospitals NHS Foundation Trust, London, United Kingdom; r2 Luton and Dunstable Hospital NHS Foundation Trust, London, United Kingdom; r3 Luton and Dunstable Hospital NHS Foundation Trust, Luton, United Kingdom; r4 University College London Hospitals NHS Foundation Trust, Luton, United Kingdom; r5 Sheffield Teaching Hospitals NHS Foundation Trust, Sheffield, United Kingdom

## Abstract

**Case report - Introduction:**

A small sub-group of COVID-19 patients develop secondary haemophagocytic lymphohistiocytosis (sHLH), a multisystem progressive hyperinflammatory syndrome characterised by fever, hepatosplenomegaly, hyperferritinaemia, cytopenia, and multiple-organ failure, which if not identified and promptly treated may be fatal. There have been isolated reports of adults developing PIMS-TS, a rare inflammatory multisystem syndrome seen in children with COVID-19 which shares common features with Kawasaki disease, toxic shock syndrome and macrophage activation syndrome / sHLH. Here we present a case of COVID-19-associated PIMS-TS in an adult complicated by frank sHLH (COV-HLH) which, after a protracted course, responded to combination immunotherapy including the IL-1 antagonist anakinra.

**Case report - Case description:**

A 22-year-old female of Nigerian-descent with sickle cell trait presented with fever, headache, sore throat, arthralgia, abdominal pain, diarrhoea/vomiting, swollen feet/legs, and macular rash on hands/forearms. A 3-day flu-like illness occurred 8 weeks earlier. Persistent pyrexia, tachycardia and hypotension required ICU admission for inotropic support. Although she briefly required oxygen, hypoxaemia was not a prominent feature. Bloods revealed CRP>200mg/L, ferritin>14,000ng/mL, raised D-Dimer, procalcitonin, Troponin-T and ALT, anaemia, lymphopenia, and neutrophilia. Computed-tomography showed mild bibasilar subpleural ground-glass changes, pelvic free fluid, and peritoneal enhancement.

As treatment for suspected COV-HLH, or connective tissue disorder, intra-venous hydrocortisone 100mg QDS was given; fever resolved and blood parameters transiently improved. Second nasopharyngeal SARS-CoV-2 RT-PCR was positive and screen for other infection and autoimmune disease negative. Echocardiography and CTA excluded coronary aneurysms although Troponin-T peak was 330ng/L.

Rapidly rising ferritin and triglycerides, falling cell counts and fibrinogen, led to a diagnosis of COV-HLH. Intra-venous anakinra 70mg (1mg/kg) BD was initiated. When pyrexia remained >40 °C, inotrope requirement persisted, cell counts fell and ferritin rose to 45,861ng/ml, anakinra was increased over 48h to 200mg BD with intra-venous methylprednisolone 1g OD x2. After 7 days anakinra was weaned to 100mg subcutaneous BD enabling discharge. Outpatient bone marrow aspirate/trephine showed reactive hyperplasia, no leukaemia or haemophagocytosis. Genomic testing showed no primary genetic cause. A week later she was readmitted with fatigue, arthralgia, pyrexia, tachycardia, haematuria, and ferritin of 23,000ng/mL (nadir 4,000ng/mL). FDG-PET showed hepatosplenomegaly with no lymphoma. Anakinra was increased to 200mg IV BD with IVIG 1mg/kg OD x2 and methylprednisolone 1g IV OD x3, then cyclosporine 1mg/kg IV BD. Fevers and haemoproteinuria resolved within 1 week and inflammatory markers fell allowing discharge on a reducing regime of subcutaneous anakinra, oral prednisolone and cyclosporine. She remained well; ferritin and FBC finally normalised >2 months after presentation.

**Case report - Discussion:**

Through the UK HLH across speciality collaboration (HASC) we are aware of only a handful of UK cases of adult presentation PIMS-TS and even fewer with frank sHLH. Our patient’s ethnic background and presentation were typical for paediatric PIMS-TS. Hence, we actively excluded coronary artery aneurysms, a key feature of the Kawasaki-type variant of PIMS-TS.

Initial COVID-19 swabs were negative as was extensive investigation for other sepsis triggers. A high clinical suspicion of COVID-19 led to the second positive swab and early recognition of sHLH. Diagnosis of HLH can be challenging due to its non-specific features and was even more difficult in critically ill patients during the peak of the pandemic, where bone marrow biopsy and cross-sectional imaging (key components of diagnostic scoring systems such as the HScore) were difficult to obtain. Persistent pyrexia, hyperferritinaemia and recognition of worsening trends in all relevant domains raised suspicion of sHLH. On initiation of anakinra, her HScore was only 118, although her illness peak was 162, well above the HASC agreed threshold of 132 for HLH diagnosis during the pandemic. She subsequently had a negative bone marrow biopsy in line with >50% of critical care patients with sHLH; a demonstration that biopsy proven haemophagocytosis is not necessary for a clinical diagnosis of sHLH. No other sHLH trigger was found.

Early recognition and intensive treatment may have contributed to the positive outcome; sHLH mortality in ICU patients can reach nearly 70%. These decisions were facilitated by early discussion with MDT members of HASC. The initial dose of 70mg IV BD and speed of wean after an effective dose was achieved were insufficient. A longer period on 400mg anakinra daily, a slower wean, plus addition of methylprednisolone, IVIG and cyclosporin appeared to aid the resolution of her relapse.

**Case report - Key learning points**
 

1. COVID-19 infection is complicated by hyperinflammatory syndromes (cytokine storm, PIMS-TS, sHLH) in a significant minority of patients. In the absence of a treatment for COVID-19, early recognition of treatable complications should be a clinical priority.
2. Adult clinicians should be aware of PIMS-TS which may rarely occur in young adults, especially those of African descent. The CDC definition extends to those aged up to 21. Cardiac aneurysms should be actively excluded in this group.
3. The challenges associated with sHLH diagnosis became more apparent during the peak of the COVID-19 pandemic where key tests were difficult to obtain. Current scoring systems are insensitive for evolving sHLH. A high index of clinical suspicion and a multidisciplinary team approach, in which rheumatologists are key, is important for early recognition and treatment. 
4. Although no other sHLH trigger was found in this case, we have seen COV-HLH patients with underlying connective tissue disorder, haematological malignancy or a primary genetic defect, which should be considered if COV-HLH patients do not respond to treatment.
5. Optimal treatment for sHLH and the hyperinflammatory syndromes associated with COVID-19 is not supported by randomised controlled trials but there is accumulating evidence for anakinra. Whilst its use in sHLH remains off-license, UK guidelines have been developed, with an emphasis on early and high dose treatment. Careful anakinra weaning regimens should be considered and patient progress regularly reviewed to avoid relapse of sHLH and subsequent readmission. Our patient also appeared to have a favourable response to corticosteroid and other combined immunosuppressive treatments including IVIG and cyclosporine. It remains to be seen if the incidence of adult-onset PIMS-TS and COV-HLH will reduce now that Dexamethasone is standard of care in adult patients with COVID-19.

